# Observations on the Deaf and Dumb

**Published:** 1859-02

**Authors:** Richard J. Dunglison


*Observations on the Deaf and Dumb.* By RICHARD J. DUNGLISON, M. D.

We have to thank the author for a copy of this valuable paper, which lately appeared in the North American Medico-Chirurgical Review. This article is instructive as throwing great light upon a subject which has been almost ignored by the profession. The statistics which are presented by Dr. Dunglison, are especially valuable. According to the article, Switzerland, in addition to being afflicted in certain districts with Cretinism, presents a most remarkable quantity of deaf mutes, one in every five hundred and three. The prevalence of this calamity in certain districts, is also noted. The author goes on to enumerate the causes, such as "Family predisposition," "Intermarriage of Relations," "Society," etc., with the causes of acquired deaf-dumbness. This is a most interesting subject, and has been ably handled by Dr. Dunglison.

